# Complete Genome Sequence of the RmInt1 Group II Intronless *Sinorhizobium meliloti* Strain RMO17

**DOI:** 10.1128/genomeA.01001-14

**Published:** 2014-10-09

**Authors:** Nicolás Toro, Francisco Martínez-Abarca, Rafael Nisa-Martínez

**Affiliations:** Grupo de Ecología Genética de la Rizosfera, Estación Experimental del Zaidín, Consejo Superior de Investigaciones Científicas (CSIC), Granada, Spain

## Abstract

We report the complete genome sequence of the RmInt1 group II intronless *Sinorhizobium meliloti* strain RMO17 isolated from *Medicago orbicularis* nodules from Spanish soil. The genome consists of 6.73 Mb distributed between a single chromosome and two megaplasmids (the chromid pSymB and pSymA).

## GENOME ANNOUNCEMENT

*Sinorhizobium meliloti* is the symbiotic partner of legumes of the genera *Medicago*, *Melilotus*, and *Trigonella*. The genomes of most of the legume symbionts are organized into several replicons and it has been proposed that these multipartite genomes and the genomic plasticity resulting from the presence of repetitive elements may be an ecological advantage, increasing the adaptive potential of these bacteria (1, 2). These repetitive elements include group II introns, a collection of self-splicing catalytic RNAs and retroelements (3, 4) widespread in bacteria (5). The genome sequences of seven *S. meliloti* strains are publicly available (6–11).

RmInt1 is a mobile group II intron that is widespread in natural populations of *Sinorhizobium meliloti* and was first described in the GR4 strain (12). This intron is generally associated with and controls the spread of the IS*Rm2011-2* insertion sequence (12–14), which is highly abundant, having been detected in all *S. meliloti* isolates tested (15). However, ~10% of *S. meliloti* isolates lack RmInt1, which is intriguing, because intron acquisition and mobility are not restricted in these isolates (16, 17).

*S. meliloti* strain RMO17 was isolated from nodules of *Medicago orbicularis* plants growing in mildly acidic soils from Riego de la Vega in León, Spain (18, 19). This RMO17 strain was confirmed to be a strain of *S. meliloti* by 16S rRNA sequencing and analyses of other taxonomic and phenotypic traits (20). Despite the presence of a large number of copies of the IS*Rm2011-2* element (12, 17, 21, 22), RMO17 is an RmInt1-less strain (17, 21).

We announce here the completion of the fully assembled and annotated genome sequence of a nonmucoid derivative of this RmInt1 intronless *S. meliloti* strain. Sequencing was performed on a GS FLX Titanium platform (Roche Diagnostics) at MACROGEN, Inc. (Korea), with both shotgun and 3 kb paired-end libraries, resulting in 180-fold genome coverage. The raw sequence data met the quality standards of the Genomes OnLine Database (GOLD) (23). GS FLX data processing was performed with Roche GS FLX software (v2.6). Assembly was achieved with the GS De Novo Assembler (v2.6), resulting in 10 scaffolds (>115 kb each; *N*_50_, 925,704 bp). Intrascaffold and interscaffold gaps were closed by the detailed observation of relevant sequencing reads with the Geneious R7 software platform (24).

The genome was annotated with the NCBI Prokaryotic Genome Annotation Pipeline. Replicon sizes and G+C contents were 3,649,532 bp (62.7%) for the chromosome, 1,610,737 bp (62.4%) for the chromid (pSymB), and 1,466,845 bp (60.4%) for the megaplasmid (pSymA). The complete genome consists of 6,136 protein-coding sequences. As in other *S. meliloti* genomes, three complete *rrn* operons were identified on the chromosome and there are 54 tRNA loci.

The complete sequence of this particular strain provides us with an opportunity to explore the evolutionary history of the presence and acquisition of group II introns and their relationship to the plasticity of complex bacterial genomes.

### Nucleotide sequence accession numbers.

The nucleotide sequences of the three replicons of the *S. meliloti* RMO17 genome have been deposited in the GenBank database under accession numbers CP009144 to CP009146.
